# P-1429. Uptake and Series Completion with Pneumococcal Conjugate Vaccine by Social Determinants of Health among Children in the United States

**DOI:** 10.1093/ofid/ofaf695.1616

**Published:** 2026-01-11

**Authors:** Amanda C Miles, Xin Zhao, Christopher G Prener, Huihua Li, Alison E Randall, Benjamin Althouse, Mark Rozenbaum, Lindsay Grant, Ronika Alexander, Paul Palmer, Maria J Tort

**Affiliations:** Pfizer, New York, NY; Genesis Research Group, Hoboken, New Jersey; Pfizer, New York, NY; Pfizer, Inc, Collegeville, Pennsylvania; Pfizer, Inc., New York, New York; Pfizer, New York, NY; Pfizer Inc., Randstad, Noord-Holland, Netherlands; Pfizer Inc., Randstad, Noord-Holland, Netherlands; Pfizer, New York, NY; Pfizer Vaccine Medical Development, Scientific & Clinical Affairs , Collegeville PA, Collegeville, PA; Pfizer, Inc, Collegeville, Pennsylvania

## Abstract

**Background:**

In the United States (US), children with unmet social and financial resources may face challenges completing well-child visits and immunizations. Pneumococcal conjugate vaccines (PCVs) are recommended as a 4-dose series at 2, 4, 6, and 12-15 months. The objective of this study was to characterize PCV uptake and series completion according to social determinants of health (SDOH) among children with commercial insurance.

**Figure:**
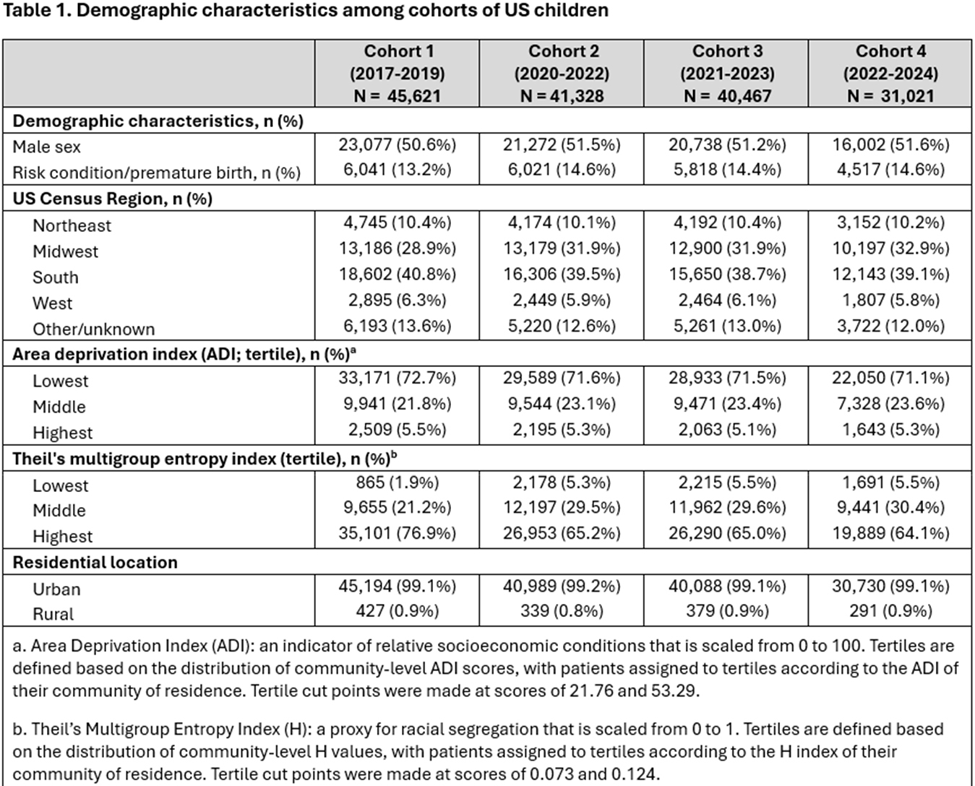

**Figure:**
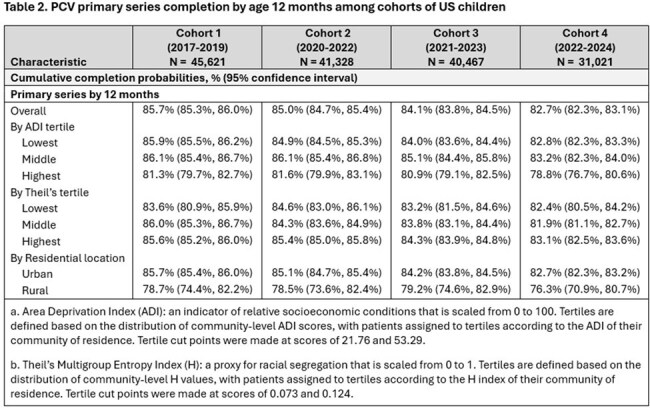

**Methods:**

Commercially insured children with continuous enrollment from 0-24 months of age were divided into four period cohorts: (Cohort 1) 2017-2019, (Cohort 2) 2020-2022, (Cohort 3) 2021-2023, and (Cohort 4) 2023-2024. PCV doses and individual-level characteristics were identified using healthcare claims from Optum’s Clinformatics DataMart®. Children were linked to community-level SDOH characteristics from the US Census Bureau’s American Community Survey and Department of Agriculture’s Urban Influence Codes using 5-digit ZIP codes. Cumulative probabilities of 3-dose primary and full series completion were evaluated using Kaplan-Meier analyses for each cohort overall and by SDOH characteristics.

**Figure:**
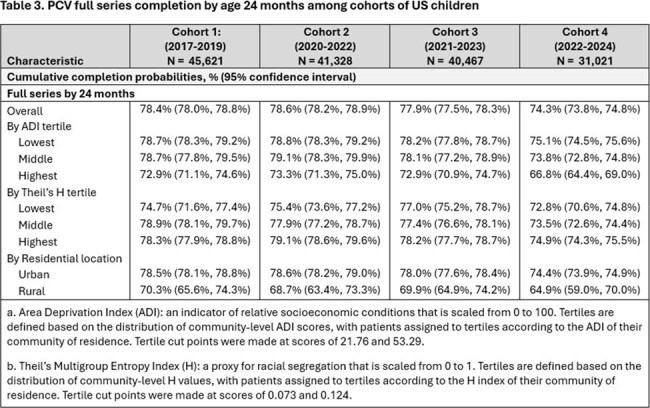

**Results:**

Between 31,021 and 45,621 children were included in each cohort. In each cohort, around half of children were male, 13% to 15% had a risk condition or premature birth. Additionally, most were from the Midwest or South regions of the US, had a low area deprivation index (ADI), a high Theil’s index, and an urban residence (Table 1). Primary series completion by 12 months and full series completion by 24 months of age was highest in Cohort 2 and lowest in Cohort 4. Compared to earlier cohorts, series completion at 12 months decreased among children in Cohorts 3 and 4 for both ADI and Theil’s index. In general, series completion was lowest in the highest ADI tertile and in the lowest Theil’s tertile. Children in rural settings had lower series completion than children in urban settings (Tables 2-3).

**Conclusion:**

PCV uptake was lowest among Cohort 4, potentially reflecting declines in uptake after the COVID-19 pandemic. Uptake was also lower among children residing in rural communities or in areas with greater deprivation. Differences in PCV uptake could contribute to disparities in disease incidence among children and could be addressed with targeted vaccination programs.

**Disclosures:**

Amanda C. Miles, MPH, Pfizer: Employee of Pfizer Inc.|Pfizer: Stocks/Bonds (Public Company) Christopher G. Prener, PhD, Pfizer: Employee|Pfizer: Stocks/Bonds (Public Company) Huihua Li, MS, MD, Pfizer Pharmaceutical: employee|Pfizer Pharmaceutical: Stocks/Bonds (Public Company) Alison E. Randall, MPH, PMP, Pfizer: Employment|Pfizer: Stocks/Bonds (Public Company) Benjamin Althouse, PhD, Pfizer, Inc: Employee|Pfizer, Inc: Stocks/Bonds (Public Company) Mark Rozenbaum, PhD, M.B.A., Pfizer: Stocks/Bonds (Public Company) Lindsay Grant, PhD, MPH, Pfizer: Employee|Pfizer: Stocks/Bonds (Private Company) Ronika Alexander, M.Ed, Pfizer, Inc.: employee|Pfizer, Inc.: Stocks/Bonds (Private Company) Paul Palmer, PhD, Pfizer Inc: Employee|Pfizer Inc: Stocks/Bonds (Public Company) Maria J. Tort, PhD, Pfizer, Inc: Stocks/Bonds (Public Company)

